# Treatment Sequencing of CDK4/6 Inhibitors in Bone-Only Hormone Receptor-Positive Metastatic Breast Cancer: Multi-Center Real-World Data Revealing a Numerical Trend Favoring Later-Line Use

**DOI:** 10.3390/cancers18172783

**Published:** 2026-08-27

**Authors:** Serkan Yıldırım, Büşra Bülbül, Bekir Ucun, İrem Turgut Yeğen, Cengiz Yılmaz, Atike Pınar Erdoğan, Ece Şahin Hafızoğlu, Erhan Gökmen, Murat Araz, Ahmet Oruç, Orhan Önder Eren

**Affiliations:** 1 Medical Oncology Department, Selçuk University Medical Faculty, 42130 Konya, Turkey; 2 İzmir City Hospital Medical Oncology, 35540 Izmir, Turkey; 3 Medical Oncology Department, Manisa Celal Bayar University Medical Faculty, 45030 Manisa, Turkey; 4 Medical Oncology Department, Ege University Medical Faculty, 35100 Izmir, Turkey; 5 Medical Oncology Department, Necmettin Erbakan University Medical Faculty, 42090 Konya, Turkey

**Keywords:** CDK4/6 inhibitors, bone metastasis, breast cancer, first-line, real-world data

## Abstract

Background: Cyclin-dependent kinase 4/6 inhibitors (CDK4/6i) combined with endocrine therapy represent the gold standard in the first-line treatment of hormone receptor (HR)-positive, HER2-negative metastatic breast cancer (MBC). However, the optimal timing of CDK4/6i initiation in patients with bone-only disease (BoD) remains a matter of clinical discussion. This multicenter retrospective study evaluated the impact of CDK4/6i treatment line (first-line vs. second-or-later-line) on progression-free survival (PFS) and overall survival (OS) in HR+/HER2− MBC patients with BoD. Methods: A total of 282 HR+/HER2− MBC patients with radiologically confirmed BoD treated across five tertiary academic centers were analyzed. All patients received bone-modifying agents (zoledronic acid or denosumab). Patients were categorized into Group 1 (first-line CDK4/6i, *n* = 193) and Group 2 (second-or-later-line CDK4/6i, *n* = 89). Baseline characteristics were recorded. Multivariable Cox proportional hazards models were adjusted for key clinicopathological variables. Results: The median PFS was 30.193 months in the first-line group and 19.253 months in the second/later-line group, with no statistically significant difference (*p* = 0.055). The median OS was 62.324 months in the first-line group and 72.00 months in the second/later-line group, which was significant in the univariate analysis (*p* = 0.036). However, this significance was not maintained in the multivariate analysis adjusting for potential confounders (*p* = 0.487). Except for strong ER positivity, baseline characteristics were balanced between groups. In the multivariate analysis, no clinical factor, including treatment line, independently and significantly impacted OS. Conclusions: In HR+/HER2− MBC patients with bone-only disease, first-line CDK4/6 inhibitor therapy demonstrates a trend toward superior PFS. After correcting for key observational biases, the later line of therapy does not independently alter OS. These findings reinforce that first-line CDK4/6 inhibitor plus endocrine therapy remains the definitive standard of care, and later-line initiation should be reserved solely for select clinical scenarios where first-line use was not feasible.

## Simple Summary

Cyclin-dependent kinase 4/6 inhibitors (CDK4/6i) combined with endocrine therapy are standard first-line treatments for hormone receptor-positive (HR+), HER2-negative metastatic breast cancer (MBC). However, the optimal sequencing of CDK4/6i for patients presenting with bone-only disease (BoD) remains unclear. In this multi-center retrospective study of 282 patients with baseline BoD, we compared progression-free survival (PFS) and overall survival (OS) between patients receiving CDK4/6i in first-line versus second- or later-line settings. While first-line use showed a trend toward longer PFS, later-line use showed a numerical trend toward longer OS, which lost statistical significance in multivariate analysis. These real-world findings highlight the need for personalized treatment sequencing in HR+/HER2− MBC patients presenting with bone-only metastasis.

## 1. Introduction

Breast cancer is the most common malignancy and a leading cause of cancer-related mortality among women [1,2,3]. The hormone receptor (HR)-positive, HER2-negative subtype constitutes approximately 75% of all breast cancers and is associated with a relatively favorable prognosis and biological course compared to other subtypes [4,5,6]. However, approximately 30% of women diagnosed with early-stage breast cancer will eventually develop metastatic disease, with bone being the most frequent site of metastasis [5,7,8]. In HR+/HER2− metastatic breast cancer (MBC), bone is the sole metastatic site in 30–60% of cases, with approximately 40% of patients presenting with bone-only disease (BoD) [9,10,11].

The incorporation of cyclin-dependent kinase 4/6 inhibitors (CDK4/6i) into the treatment paradigm of HR+/HER2− MBC has significantly improved disease management. The landmark PALOMA, MONALEESA, and MONARCH phase 3 trials demonstrated that the combination of CDK4/6i with endocrine therapy significantly prolongs progression-free survival (PFS) in the first-line setting, establishing this regimen as the standard of care [12,13,14]. Currently, three CDK4/6i agents—palbociclib, ribociclib, and abemaciclib—are approved for the treatment of HR+/HER2− MBC [15].

Nevertheless, the efficacy of CDK4/6i in distinct patient subgroups remains a subject of debate. In particular, the magnitude of PFS benefit from CDK4/6i in patients with BoD appears variable [16]. A retrospective study by Mardani et al. reported a significantly shorter median PFS in the BoD group compared to patients with bone and visceral metastases (13.1 vs. 19.6 months; *p* = 0.01) [17]. In their multivariate analysis, BoD was identified as an independent risk factor for adverse PFS (HR = 1.62; 95% CI: 1.04–2.54) [17]. Preclinical evidence also suggests that CDK4/6i may be less effective within the bone microenvironment than in visceral metastases, potentially due to the presence of immunosuppressive macrophages [17]. In contrast, subgroup analyses of the MONALEESA and MONARCH trials showed that CDK4/6i were effective in the BoD population [13,14].

Consequently, the optimal sequencing of CDK4/6i therapy—specifically whether every patient with indolent, bone-limited disease mandates upfront CDK4/6i or whether endocrine monotherapy followed by second-line CDK4/6i provides equivalent long-term OS—remains a critical clinical question. The randomized phase 3 SONIA trial recently reported that sequencing endocrine monotherapy first followed by CDK4/6i at progression yielded comparable OS and quality of life compared with upfront first-line CDK4/6i use in the overall advanced HR+/HER2− population [18]. However, like many other studies, this trial included all metastatic patients, and comprehensive analyses focusing specifically on the BoD subgroup remain limited.

The aim of this multi-center retrospective study was to compare OS and PFS between first-line and second-or-later-line use of CDK4/6i in patients with HR+/HER2− MBC and BoD.

## 2. Materials and Methods

### 2.1. Study Design and Patient Selection

This multi-center retrospective cohort study evaluated 282 patients with HR+/HER2− MBC who presented with baseline bone-only disease (BoD) and received CDK4/6i therapy between January 2017 and December 2024 across five academic and tertiary oncology centers in Türkiye: Selçuk University Medical Faculty, Ege University Medical Faculty, Manisa Celal Bayar University Medical Faculty, Necmettin Erbakan University Medical Faculty, and İzmir City Hospital. All patients were receiving bone-modifying agents (zoledronic acid or denosumab) for their bone metastases. Patients were divided into two groups based on the line of CDK4/6i therapy:

Group 1 (First-Line): Patients who received CDK4/6i in combination with endocrine therapy as their initial first-line systemic regimen for metastatic disease (*n* = 193).

Group 2 (Second-or-Later-Line): Patients who received endocrine therapy or chemotherapy as first-line therapy and subsequently initiated CDK4/6i therapy in the second- or later-line setting upon disease progression (*n* = 89).

Inclusion criteria were (1) histopathologically confirmed HR-positive (ER and/or PR ≥ 1%) and HER2-negative (IHC 0, 1+, or IHC 2+ with negative ISH) adenocarcinoma of the breast; (2) radiologically documented bone-only metastatic disease at the time of baseline metastatic evaluation, confirmed by computed tomography (CT), magnetic resonance imaging (MRI), bone scintigraphy, or 18F-FDG PET/CT; (3) receipt of at least one cycle of CDK4/6i therapy (palbociclib or ribociclib); and (4) available medical and longitudinal survival follow-up records.

### 2.2. Clinical Variables and Metastatic Site Definitions

Exclusion criteria were the presence of visceral or central nervous system metastases at baseline, concurrent second primary malignancies, or HER2-positive status.

Definition of Baseline vs. Subsequent Progression Involvement: Metastatic site status (bone-only disease vs. visceral involvement) was defined strictly as a baseline characteristic prior to initiating CDK4/6 inhibitor therapy. While all included patients strictly met the criteria for baseline bone-only metastasis, subsequent disease progression during the treatment continuum was not restricted to skeletal tissue and could involve visceral organs, soft tissue, and/or bone. Patients were permitted to have regional lymph node metastases (not classified as distant metastases) alongside bone metastases. Demographic, clinical, and pathological characteristics were recorded using a standardized data form across all centers. The parameters evaluated included: age (>65 years, <35 years), pathological subtype (invasive ductal carcinoma [IDC], invasive lobular carcinoma [ILC], other), metastatic presentation (de novo vs. systemic recurrence), ER expression level (>90%, 50–90%, <50%), PR status (positive, negative), HER2 status (HER2-negative, HER2-low), menopausal status (postmenopausal, premenopausal), type of CDK4/6i (ribociclib, palbociclib), and line of CDK4/6i therapy (first-line vs. second-line or later).

### 2.3. Study Endpoints

Overall Survival (OS): Defined as the time from the date of MBC diagnosis (for patients in the second-or-later-line group, the date of MBC diagnosis was used) to death from any cause.

Progression-Free Survival (PFS): Defined as the time from the initiation of CDK4/6i therapy to documented radiological/clinical progression or death from any cause.

### 2.4. Statistical Analysis

Statistical analyses were performed using SPSS (IBM SPSS Statistics, version 26.0). Categorical variables were presented as frequencies and percentages, and continuous variables as medians with interquartile ranges (IQR). Comparisons between groups for categorical variables were performed using the chi-square test or Fisher’s exact test, as appropriate.

PFS and OS were estimated using the Kaplan–Meier method and compared using the log-rank test. Univariate and multivariate analyses were performed using the Cox proportional hazards regression model. Variables with a *p*-value < 0.20 in the univariate analysis or considered clinically relevant (age, pathological subtype, metastatic pattern, ER level, PR status, menopausal status, and CDK4/6i type) were included in the multivariate model. A *p*-value < 0.05 was considered statistically significant.

### 2.5. Ethics Approval

The study was conducted in accordance with the principles of the Declaration of Helsinki and was approved by the Ethics Committee of Selçuk University Faculty of Medicine (Protocol code: 2025/20, Date of approval: 14 January 2025). Local ethics committee approvals were obtained from all participating centers.

## 3. Results

### 3.1. Patient and Treatment Characteristics

The clinicopathological characteristics of the 282 included patients, stratified by CDK4/6i treatment line, are summarized in Table 1. The median age was 58 years (range: 28–84), with 71.3% (n = 201) of patients being under 65 years of age. The majority of patients (85.5%, n = 241) had invasive ductal carcinoma. All patients received bone-modifying agents (zoledronic acid or denosumab) for their bone metastases.

The two cohorts were well balanced with respect to age distribution, histological subtype, de novo versus recurrent presentation, PR status, HER2 status, menopausal status, and type of CDK4/6 inhibitor (*p* > 0.05 for all comparisons; Table 1). However, the proportion of patients with strong ER positivity (≥90%) was significantly higher in the second/later-line group (53.9% vs. 39.3%, *p* = 0.002).

### 3.2. Survival Analyses

**Figure 1:**
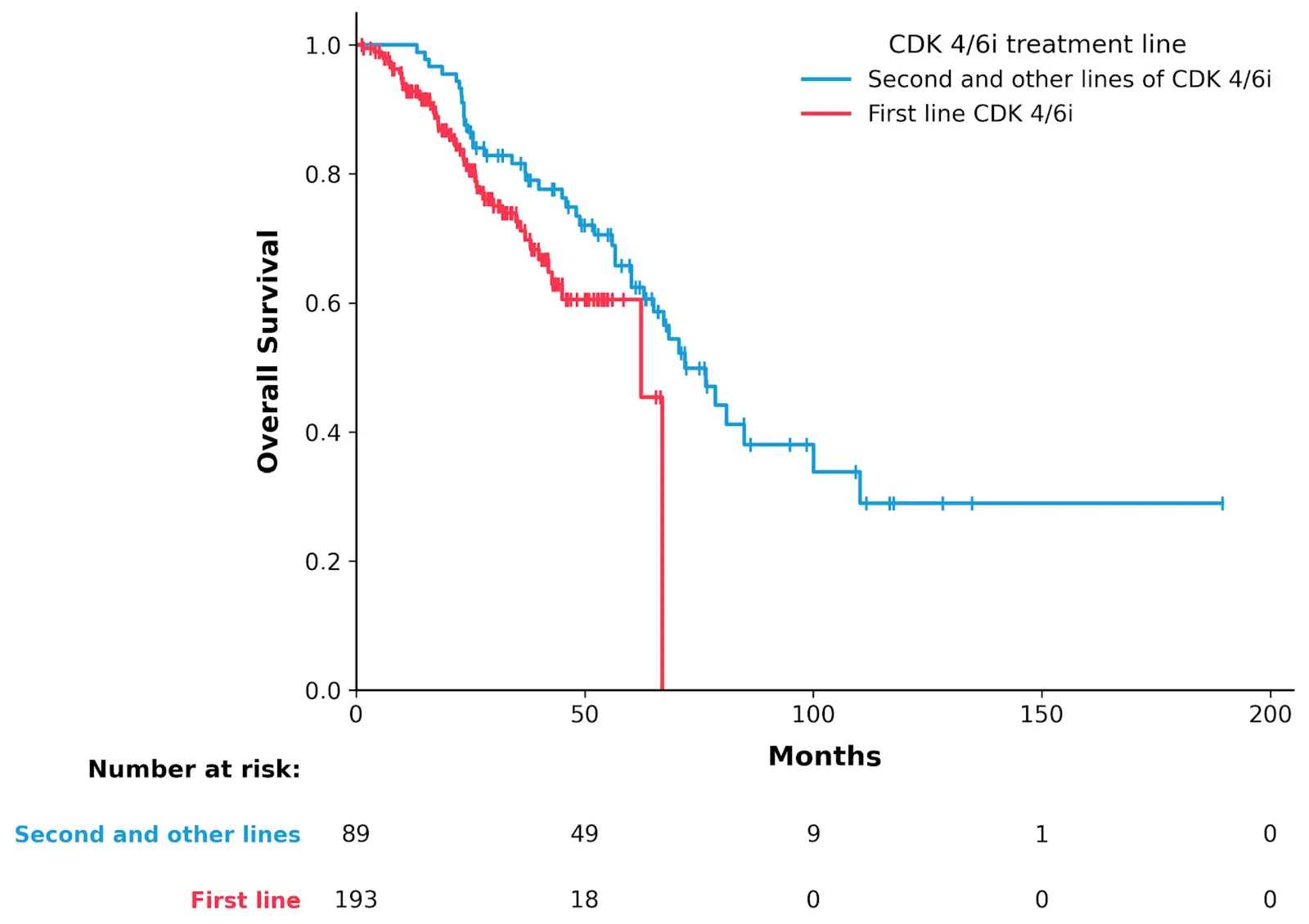
Kaplan–Meier curves comparing OS between patients receiving CDK4/6 inhibitors as first-line therapy (blue line, *n* = 89) versus second or later lines (red line, *n* = 193) in hormone receptor-positive, HER2-negative metastatic breast cancer with bone-only disease. The log-rank test showed a statistically significant difference between groups (*p* = 0.036). Median OS was 62.324 months for the first-line group and 72.00 months for the second/later-line group.

At a median follow-up of 23.83 months (IQR: 14.2–38.1 months; 27.76 months for the second/later-line group and 21.97 months for the first-line group), 47 deaths occurred in the first-line cohort and 41 deaths in the second/later-line cohort. For the primary endpoint of OS, the median OS was 62.32 months in the first-line group versus 72.00 months in the second/later-line group. In univariate Cox regression analysis, second- or later-line CDK4/6i therapy was associated with a statistically significant prolongation of OS compared to upfront first-line therapy (*p* = 0.036; Table 2, Figure 1). For PFS, the median PFS was 30.193 months in the first-line group and 19.253 months in the second/later-line group (*p* = 0.055).

**Figure 2:**
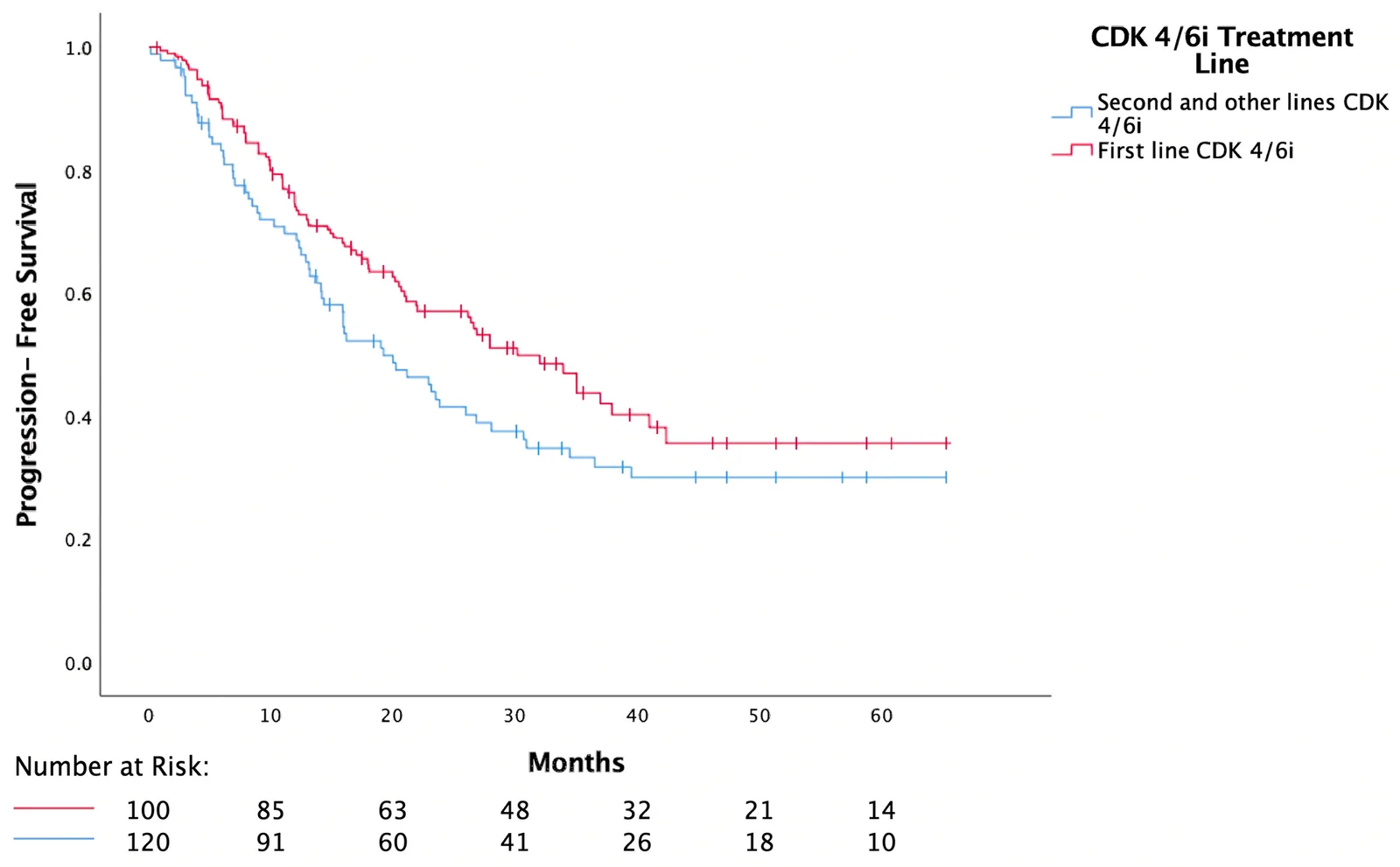
Kaplan–Meier curves comparing PFS between patients receiving CDK4/6 inhibitors as second or later lines therapy (blue line, n = 89) versus first line (red line, n = 193) in hormone receptor-positive, HER2-negative metastatic breast cancer with bone-only disease. The log-rank test showed borderline significance between groups (*p* = 0.055). Median PFS was 30.193 months for the first-line group and 19.253 months for the second/later-line group.

In the univariate Cox regression analysis, receiving CDK4/6i in the second or later line was associated with a statistically significant OS benefit compared to first-line use (*p* = 0.036) (Table 2, Figure 1). No statistically significant difference was observed for PFS (*p* = 0.055) (Table 2, Figure 2).

In the multivariate analysis, which adjusted for clinicopathological factors including age, pathological subtype, metastatic pattern, ER/PR status, menopausal status, and CDK4/6i type, the effect of treatment line on OS lost its statistical significance (*p* = 0.078) (Table 2).

When evaluating the impact of other clinicopathological variables on OS, no factor reached statistical significance in either the univariate or multivariate analyses. These included age < 35 years (univariate *p* = 0.965, multivariate *p* = 0.648), age > 65 years (univariate *p* = 0.862, multivariate *p* = 0.503), ER ≥ 90% (univariate *p* = 0.710, multivariate *p* = 0.777), HER2-low status (univariate *p* = 0.820, multivariate *p* = 0.915), menopausal status (univariate *p* = 0.136, multivariate *p* = 0.068), metastatic pattern (univariate *p* = 0.123, multivariate *p* = 0.327), pathological subtype (univariate *p* = 0.691, multivariate *p* = 0.543), PR status (univariate *p* = 0.474, multivariate *p* = 0.379), and type of CDK4/6i (univariate *p* = 0.434, multivariate *p* = 0.407).

### 3.3. Subgroup Analyses

In pre-specified subgroup analyses (age groups, ER/PR status, metastatic pattern, CDK type), no significant interaction was found for the effect of CDK4/6i treatment line on OS (all interaction *p*-values > 0.05).

## 4. Discussion

In this multi-center real-world study encompassing 282 patients with HR+/HER2− metastatic breast cancer presenting with baseline bone-only disease, the sequencing of CDK4/6 inhibitor therapy (upfront first-line vs. second- or later-line) did not demonstrate an independent, statistically significant impact on overall survival in multivariate analysis (*p* = 0.078). Although a statistically significant OS advantage was observed for second/later-line use in the univariate analysis (*p* = 0.036), this significance was lost after adjusting for potential confounders in the multivariate analysis (*p* = 0.078). For progression-free survival (PFS), a numerical advantage was noted for the first-line group, but this difference did not reach statistical significance.

The loss of significance in the multivariate analysis strongly suggests that the observed OS benefit was largely driven by confounding factors. Patients receiving CDK4/6i in the second or later line inherently represent a more favorable prognostic group, as they had to survive long enough to complete first-line therapy and progress to subsequent treatment. Conversely, patients who did not receive first-line CDK4/6i and were lost to early progression or death were not included in this analysis. This introduces both “survival bias” and “immortal time bias,” potentially leading to falsely positive results in favor of the later-line group [19]. The disappearance of significance in the multivariate analysis supports the presence of such bias.

Notably, none of the other clinicopathological factors evaluated in this study were found to be significant predictors of OS in either univariate or multivariate analyses. These factors included age < 35 years (*p* = 0.965 and *p* = 0.648), age > 65 years (*p* = 0.862 and *p* = 0.503), ER ≥ 90% (*p* = 0.710 and *p* = 0.777), HER2-low status (*p* = 0.820 and *p* = 0.915), menopausal status (*p* = 0.136 and *p* = 0.068), metastatic pattern (*p* = 0.123 and *p* = 0.327), pathological subtype (*p* = 0.691 and *p* = 0.543), PR status (*p* = 0.474 and *p* = 0.379), and type of CDK4/6i (*p* = 0.434 and *p* = 0.407). The borderline significance of menopausal status (*p* = 0.068) in the multivariate analysis warrants further investigation in larger cohorts. Interestingly, while our study suggests that premenopausal patients receiving CDK4/6i in later lines may have had longer OS, the phase 3 SONIA trial reported in a subgroup analysis that premenopausal patients derived more benefit from first-line therapy [18]. This discrepancy is likely attributable to the smaller sample size and residual confounding in our study.

Our findings are consistent with existing real-world data. A multi-center study from Poland involving 1511 HR+/HER2− MBC patients found no significant differences in PFS, PFS2, or OS between first-line and second-line CDK4/6i use [20]. Similarly, a real-world study from Italy reported no significant impact of CDK4/6i treatment line (first vs. second) on survival in patients with bone metastases [21].

Preclinical data suggest that CDK4/6i may be less effective in the bone microenvironment compared to visceral sites [17]. In a bone colonization model, ribociclib failed to significantly impact tumor burden and did not reduce the number of Arg1+ immunosuppressive macrophages, pointing towards treatment resistance and immune suppression within the bone niche [17]. Furthermore, it has been proposed that CDK4/6i might promote osteoclast activation via the TGF-β signaling pathway and facilitate tumor cell retention in the bone marrow [22]. The more limited PFS benefit observed with CDK4/6i in BoD patients compared to those with visceral metastases supports this hypothesis [8,9]. In our study, the numerically longer PFS observed in the first-line group (30.193 vs. 19.253 months) suggests that earlier initiation of CDK4/6i could contribute to progression-free survival, although this difference was not statistically significant.

High ER expression (≥90%) has been reported as an independent prognostic factor associated with greater OS benefit from CDK4/6i therapy [23]. In our study, the second/later-line group had a significantly higher proportion of patients with strong ER positivity (53.9% vs. 39.3%, *p* = 0.002). Although ER expression was adjusted for in the multivariate analysis, this underlying biological difference between the groups may have influenced the results. The generally better prognosis of patients with high ER expression could partly account for the OS advantage observed in the second/later-line group.

The use of bone-modifying agents (denosumab, zoledronic acid) in patients with bone metastases is known to reduce skeletal-related events and may contribute to improved survival [24,25,26]. In our study, all patients received such agents, and their distribution was balanced between the groups.

A critical methodological consideration in analyzing retrospective oncology cohorts is immortal time bias and confounding by indication. In unadjusted comparisons, patients receiving later-line therapies may falsely exhibit favorable survival because they survived long enough to undergo prior therapeutic lines and demonstrate an indolent tumor phenotype. By establishing time endpoints (PFS and OS) measured directly from the initiation of CDK4/6 inhibitor therapy, our adjusted multivariable analysis confirms that the observed univariate survival trend for later-line therapy was non-causal and largely driven by immortal time bias and biological selection [19]. Furthermore, confounding by indication remains a substantial limitation in observational studies comparing treatment lines. In routine clinical practice, treatment choices are strongly driven by unmeasured clinical variables such as ECOG performance status, baseline symptomatic disease burden, skeletal-related events (SREs), exact number/location of bone lesions, prior duration of adjuvant endocrine response, and specific disease kinetics. Patients receiving single-agent endocrine therapy prior to CDK4/6i may represent an indolent oligometastatic subgroup. In contrast, patients in de novo or aggressive settings are prioritized for upfront doublet therapy. In selected clinical scenarios, such as de novo presentation or oligometastatic burden, multimodal approaches including loco-regional therapy or primary tumor resection after systemic control have also been investigated with variable clinical outcomes [27]. Another important factor is treatment heterogeneity. In our real-world cohort, patients received either palbociclib or ribociclib, primarily combined with aromatase inhibitors or fulvestrant. Differences in endocrine partners, prior adjuvant exposure, treatment duration, and dose reductions reflect real-world clinical complexity. Notably, abemaciclib was not utilized in this patient series, which may limit direct generalizability across all available CDK4/6 inhibitors. Finally, the interpretation of median survival estimates in cohorts with short median follow-up (23.8 months) requires caution. Due to the favorable biological nature of HR+/HER2− bone-only disease, event rates (deaths) remain low within the initial follow-up window, rendering median OS point estimates sensitive to censoring. Therefore, report of hazard ratios with 95% confidence intervals and event counts provides a more reliable metric than unadjusted median Kaplan–Meier estimates.

The strengths of this study include its multi-center design, which enhances the heterogeneity and representativeness of the patient population by including five large reference centers from different regions of Turkey. The focus on a purely BoD population eliminates confounding by visceral metastases. The detailed recording and adjustment for clinicopathological parameters is another strength.

Limitations of this study include its retrospective design, which inherently introduces selection bias and potential for missing data. We were unable to account for potentially important confounders such as ECOG performance status, comorbidities, duration of prior endocrine therapy response, specific type and duration of bone-modifying agents, and prior radiotherapy. The median follow-up period of 23.83 months may be insufficient to fully capture long-term OS outcomes. Additionally, the absence of patients treated with abemaciclib limits the generalizability of our findings to all CDK4/6i agents.

## 5. Conclusions

In conclusion, this multicenter real-world study demonstrates that first-line use of CDK4/6 inhibitors in HR+/HER2− metastatic breast cancer with bone-only disease provides a numerical PFS benefit over later-line use. When accounting for immortal time bias and confounding factors through multivariable analysis, the line of CDK4/6i therapy does not yield an independent OS difference. These findings do not support delaying CDK4/6 inhibitor treatment or interpreting retrospective equivalence as justification for second-line deferral. Upfront CDK4/6 inhibitor plus endocrine therapy remains the definitive standard of care for patients with bone-only MBC. Later-line CDK4/6 inhibitor administration should be considered only when first-line combination therapy is medically contraindicated or unfeasible.

Prospective, randomized controlled trials and larger-scale real-world data analyses are necessary to definitively determine the optimal sequencing of CDK4/6i in BoD patients and to identify predictive biomarkers of treatment response. Furthermore, a better understanding of resistance mechanisms to CDK4/6i within the bone microenvironment is essential for developing more effective treatment strategies for this patient population.

## Figures and Tables

**Table 1:** Clinicopathological Characteristics of Patients.

Parameter	Subgroup	Second/Later-Line (n = 89)	First-Line (n = 193)	*p*-Value
Age > 65 years	No	67 (75.3%)	133 (68.9%)	0.281
	Yes	22 (24.7%)	60 (31.1%)	
Age < 35 years	No	87 (97.8%)	186 (96.4%)	0.537
	Yes	2 (2.2%)	7 (3.6%)	
Pathology	Invasive Ductal	72 (80.9%)	169 (87.6%)	0.186
	Invasive Lobular	10 (11.2%)	19 (9.8%)	
	Other	7 (7.9%)	5 (2.6%)	
Metastatic Pattern	De Novo	51 (57.3%)	107 (55.4%)	0.765
	Systemic Recurrence	38 (42.7%)	86 (44.6%)	
ER Strong Positive	No	52 (58.4%)	74 (38.3%)	0.002
	Yes (>90%)	35 (39.3%)	104 (53.9%)	
ER < 50%	No	79 (88.8%)	163 (84.5%)	0.340
	Yes	8 (9.0%)	15 (7.8%)	
PR	Negative	20 (22.5%)	33 (17.1%)	0.287
	Positive	67 (75.3%)	145 (75.1%)	
HER2 Status	HER2-negative	51 (57.3%)	89 (46.1%)	0.080
	HER2-low	38 (42.7%)	103 (53.4%)	
Menopausal Status	Premenopausal	29 (32.6%)	65 (33.7%)	0.855
	Postmenopausal	60 (67.4%)	128 (66.3%)	
CDK Type	Ribociclib	65 (73.0%)	136 (70.5%)	0.658
	Palbociclib	24 (27.0%)	57 (29.5%)	

Data are presented as n (%). *p*-values were calculated using the chi-square test.

**Table 2:** Univariate and Multivariate Cox Regression Analyses for Overall Survival (OS).

Variable	Category (Reference)	Univariate *p*	Multivariate *p*
CDK4/6i treatment line	2nd/later vs. 1st line	0.036	0.078
Age < 35 years	Yes vs. No	0.965	0.648
Age > 65 years	Yes vs. No	0.862	0.503
ER ≥ 90%	Yes vs. No	0.710	0.777
HER2-low	Yes vs. No	0.820	0.915
Menopausal status	Postmenopausal vs. Premenopausal	0.136	0.068
Metastasis pattern	De novo vs. Systemic recurrence	0.123	0.327
Pathological subtype	Invasive lobular/other vs. Invasive ductal	0.691	0.543
PR status	Positive vs. Negative	0.474	0.379
CDK4/6i type	Palbociclib vs. Ribociclib	0.434	0.407

Abbreviations: ER, estrogen receptor; PR, progesterone receptor; OS, overall survival. The multivariate model was adjusted for age, pathological subtype, metastasis pattern, ER status, PR status, menopausal status, and CDK4/6i type.

## Data Availability

The data presented in this study are available on request from the corresponding author.
